# CD320 Receptor and Vitamin B12 as Potential Targets for Anti-Cancer Therapy

**DOI:** 10.3390/ijms26125652

**Published:** 2025-06-12

**Authors:** Ainur Tolymbekova, Larissa Lezina

**Affiliations:** School of Medicine, Nazarbayev University, Astana 010000, Kazakhstan; ainur.tolymbekova@nu.edu.kz

**Keywords:** CD320, transcobalamin receptor, TCblR, vitamin B12, targeted drug delivery, anti-cancer therapy, selective targeting

## Abstract

Despite the development of a wide plethora of different anticancer agents, most of them are not used for patient treatment due to adverse effects caused by untargeted cytotoxicity. To prevent this unwanted toxicity, it is necessary to develop therapies discriminating between healthy and cancerous cells. One possible method is to target proteins overexpressed in cancer but not in normal cells. CD320 is a receptor responsible for the uptake of the transcobalamin-bound fraction of vitamin B12 (cobalamin), which is necessary for DNA synthesis, and thus, cell proliferation. CD320 was shown to be overexpressed in many cancers and its potential role as an early cancer biomarker was confirmed in several studies. Consequently, CD320 may represent a promising anti-cancer therapy target. This review summarizes the current advances and perspectives of anti-cancer CD320 targeting therapy, including therapeutic conjugates of vitamin B12, CD320-specific antibodies and nanobodies, nanoparticles loaded with cytotoxic drugs, porphyrin, and the potential of targeted CD320 therapy in attenuation of tumor tissues. Given the growing interest in CD320 as a novel target for anti-cancer therapy, further in vivo studies are required for the investigation of CD320 targeting effects on systemic cytotoxicity.

## 1. Introduction

A successful anti-cancer therapy needs to discriminate between tumor and normal tissue to minimize any adverse effects on the patient [1]. Therefore, targeting the proteins and pathways upregulated in cancer, but not in normal cells, seems to be a viable strategy for therapeutic intervention. Solid tumor cells require a significant amount of nutrients and energy to survive and proliferate, leading to an overexpression of receptors, proteins, and molecules, essential for tumor progression [1]. CD320 is a cell surface receptor that delivers the transcobalamin-bound fraction of vitamin B12 inside the cell [2,3]. It was shown to be overexpressed in many cancers, including leukemia, lymphoma, breast, ovarian, lung, bone, thyroid, colon, prostate, liver, melanoma, testicular, and brain cancer, thus making it a good target for cancer therapy [1,2,4,5,6,7,8]. This review aims to discuss the perspectives of the CD320 receptor as a potential target for selective anti-cancer therapy.

### 1.1. Vitamin B12 Metabolism

**Figure 1:**
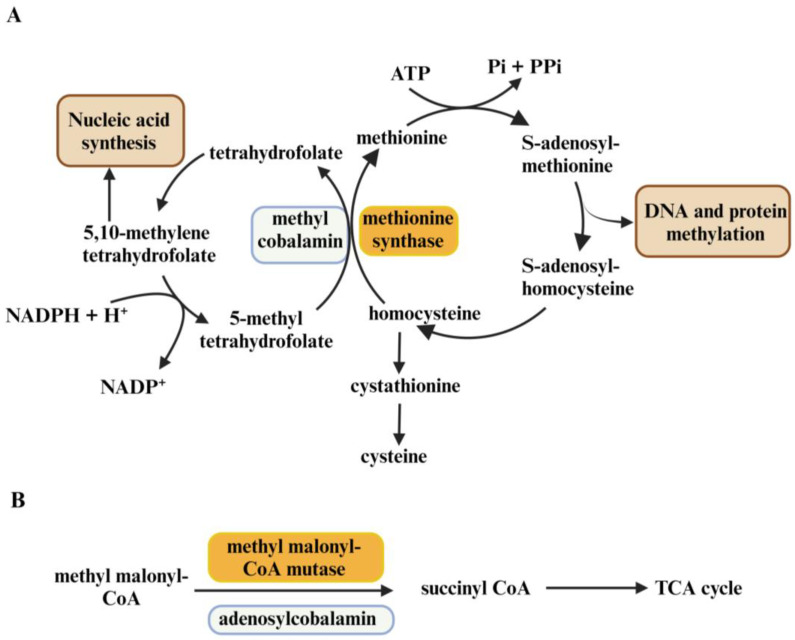
VB12 is a coenzyme for methionine synthase and methyl malonyl-CoA mutase enzymes. (**A**). Methionine synthase uses methylcobalamin as a coenzyme to produce methionine from homocysteine, which results in the formation of S-adenosyl-methionine essential for DNA and protein methylation. Tetrahydrofolate, formed during the formation of methionine, is further used for nucleic acid synthesis. (**B**). Methyl malonyl-CoA mutase uses adenosylcobalamin as a coenzyme to produce succinyl-CoA from methyl malonyl-CoA, which is further used in the TCA cycle. Created in Biorender (https://app.biorender.com/illustrations/676bf15fb102c9ede216f367?slideId=f776fcac-a95d-4495-8b4b-f6707043f0ba, accessed on 29 April 2025).

Vitamin B12 (VB12), also called cobalamin (Cbl), is a water-soluble vitamin. It is a cofactor for two enzymes in humans: methionine synthase (MS) and methylmalonyl-CoA mutase (MCM) (Figure 1) [9,10,11]. MS catalyzes the formation of methionine via the methylation of homocysteine involved in methyltransferase reactions, nucleotides, and cysteine synthesis (Figure 1) [11]. MCM converts methylmalonyl-CoA to succinyl-CoA crucial for the TCA cycle (tricarboxylic acid cycle), ATP, fatty acid and amino acid metabolism (Figure 1) [10,11,12]. Despite low daily requirements of VB12 (2.5 ug/day), its deficiency results in megaloblastic anemia, cognitive dysfunction, cardiovascular symptoms, ataxia, paresthesia, spinal cord and peripheral nerve demyelination, and methylmalonic aciduria [10,11,13,14]. A recent analysis of VB12 levels in patient serum samples revealed its positive correlation with advanced stages of breast, colon, and lung cancers [15,16]. This can probably be explained by the important role of VB12 in DNA synthesis and energy metabolism, both of which are essential for actively proliferating cancer cells.

Humans rely solely on the dietary uptake of VB12 [14]. The absorption and uptake of VB12 by cells are coordinated by a complex of proteins [11]. Upon release into the bloodstream, VB12 becomes bound to its transporter proteins haptocorrin (transcobalamin I) and transcobalamin (TC), also referred to as transcobalamin II [17]. Most commonly, circulating VB12 is bound to haptocorrin, and only 20–30% of VB12 in the blood is TC-bound [18,19]. Haptocorrin bound to VB12 is internalized by hepatocytes via asialoglycoprotein receptor, while transcobalamin–VB12 complex (TC-VB12) is recognized by megalin and CD320 receptors [11]. Megalin expressed on proximal renal tubules is responsible for the renal absorption of VB12, while CD320 (also referred to as transcobalamin II receptor or TCblR) is ubiquitously expressed in all cell types and responsible for the cellular uptake of VB12, except for in liver cells [11,20,21,22].

### 1.2. CD320 Gene and Receptor

**Figure 2:**
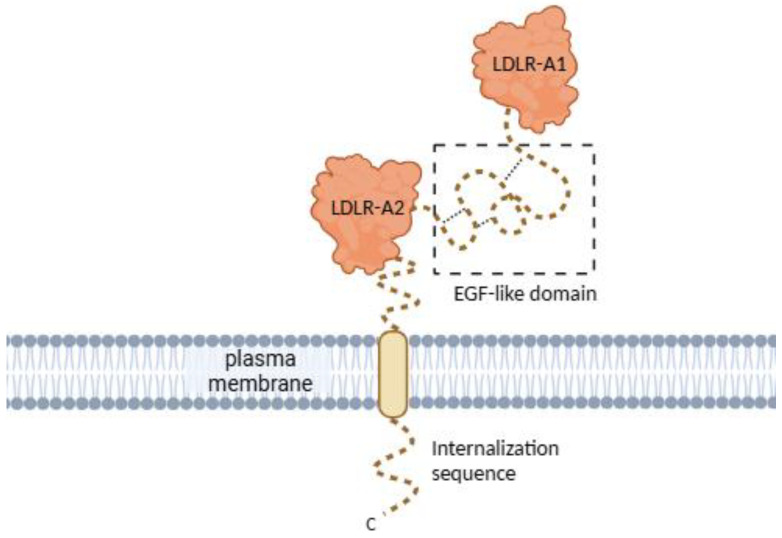
CD320 receptor structure. CD320 consists of two LDLR-A domains joined by an EGF-like domain, a cytoplasmic internalization sequence, and a transmembrane domain. The flexible regions (C-terminal internalization sequence and EGF-like domain) of CD320 were either truncated or not resolved in the crystal structures, thus depicted schematically. Created in Biorender (https://app.biorender.com/illustrations/670ca364f8ad8511e6dc2a4b?slideId=2d2ce726-1bf6-4eee-8618-3918953cd24a, accessed on 29 April 2025).

The human *CD320* gene is located on chromosome 19p13.2. It contains five coding exons and four introns [2,13]. The gene encodes a protein made of 282 amino acids, which form the extensive extracellular domain, single transmembrane domain, and short cytoplasmic domain (Figure 2) [2,13]. The extracellular domain consists of two heavily glycosylated LDLR (low-density lipoprotein receptor) type A domains responsible for the recognition of, and binding to, the TC-VB12 complex [13,23]. The cytoplasmic domain is expected to be involved in the internalization of CD320 bound to TC-VB12 [20].

### 1.3. CD320 Expression Is Cell-Cycle Associated

The expression of CD320 is cell-cycle dependent [24,25,26,27]. CD320 receptor expression peaks during the log phase of growth and depends on the initial seeding density of cells in vitro [24]. During an actively dividing phase, the plasma membrane content of CD320 reaches about 2000–6000 receptors per cell, which upon differentiation drops to less than 300 receptors, indicating the importance of CD320 in cell proliferation [26,27,28]. Moreover, *CD320* gene promoter activity was cell cycle-associated [29]. The transcription factors identified to bind the CD320 promoter regulatory elements, including MZF-1/RREB1 (activating factors), C/EBP/HNF-3β, and AP-1 (inhibiting factors), are found to be involved in cell proliferation, growth, and differentiation [29].

The cyclic expression of CD320 was hypothesized to be correlated to the demand for the production of tetrahydrofolate, which is necessary for DNA synthesis and requires an optimal supply of VB12 [9,10,11,13,24]. The correlation between the expression of CD320 and DNA synthesis was indeed confirmed [28].

### 1.4. Cells Rely on the Expression of New CD320 Receptors

Mammalian CD320 has a high affinity and specificity for the TC-VB12 complex, the binding of which initiates clathrin-mediated endocytosis [24]. Some studies argue that, upon internalization, the receptor becomes recycled back to the plasma membrane [27,30]. However, a loss of CD320-GFP and TC-dsRed signals after the incubation of HEK293 cells with TC-VB12 labeled complexes points to the lysosomal degradation of both CD320 and TC, resulting in the release of VB12 [24]. Furthermore, the plasma membrane content of CD320 was shown to depend on new protein synthesis rather than endosomal recycling [28]. Thus, to meet the high demand for VB12, cancer cells need to overexpress the CD320 receptor [1,4,5,6].

## 2. Potential Cancer Treatment Strategies Based on CD320 Targeting

**Figure 3:**
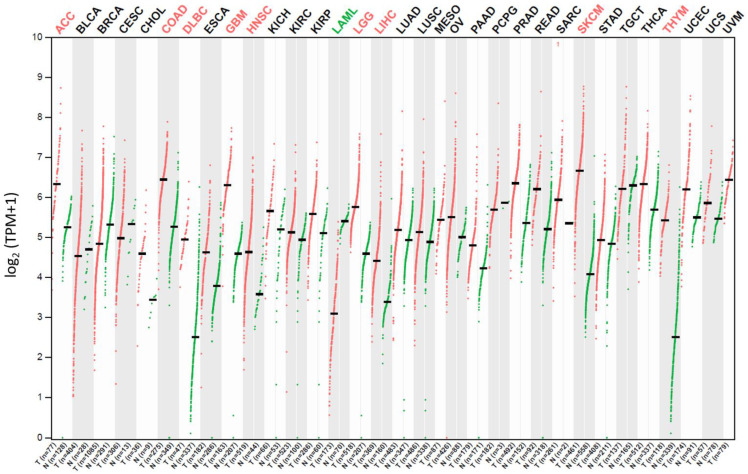
The CD320 gene expression profile in tumor vs. normal samples in different cancer patient tissues as indicated on top of the graph. The expression unit is log_2_(transcription per million (TPM) + 1). Green and red colored dots represent data for normal and tumor tissue samples, respectively. One dot corresponds to one patient sample. Data were retrieved from the Gene Expression Profiling Interactive Analysis 2 (GEPIA2) database [33,34].

Targeted drug delivery reduces the systemic toxicity of the drug via discrimination between normal and cancerous cells and allows for the delivery of higher doses of the drug to the tumor site while reducing its overall load [13,31]. The increased expression of CD320 in many cancers (leukemia, lymphoma, breast, ovarian, lung, bone, thyroid, colon, prostate, liver, melanoma, testicular, and brain cancer) and cell cycle-associated expression patterns seem to make CD320 a good potential target for cancer treatment (Figure 3) [1,2,4,5,6,7,8,24,32].

**Figure 4:**
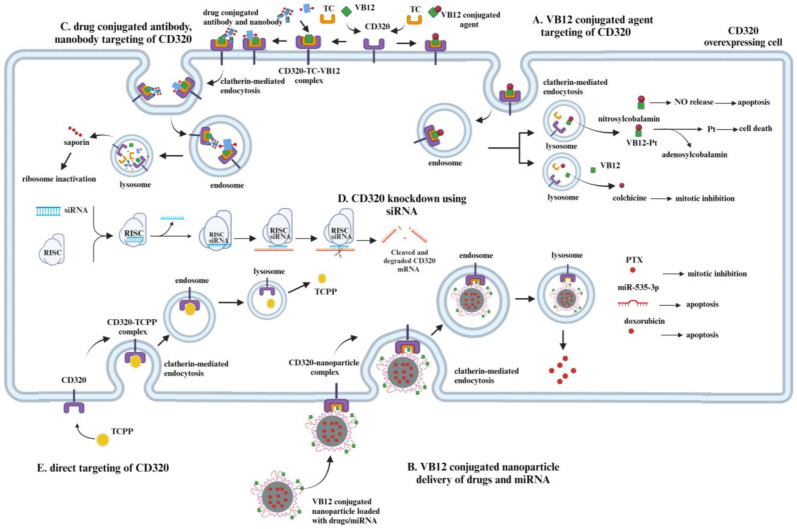
Different methods of targeting a CD320 overexpressing cell. (**A**). VB12 conjugated chemical agents can be used to enter cells via CD320 receptors, overexpressed in some cancer cells. After entering the cell via clathrin-mediated endocytosis, the release of the agent takes place in the lysosome, triggered by a pH change. Pt and NO release takes place only after the release from the lysosome via enzymatic reduction in VB12, while colchicine is released from the VB12–colchicine complex upon hydrolysis in the lysosome. (**B**). VB12-conjugated nanoparticles can also be used to deliver different means of treatment to CD320 overexpressing cells. The PTX release inside the cell, depending on its concentration, will result in mitotic inhibition. miR-535-3p delivery to cells will induce apoptosis via the inhibition of the apoptosis repressor. Doxorubicin initiates apoptosis via induction of DNA damage. (**C**). CD320-targeting antibodies and nanobodies conjugated with drugs are other methods of targeting CD320-overexpressing cells. Upon the internalization of antibodies and nanobodies conjugated to the drug and their degradation in the lysosome, the release of a drug takes place. In that case, released saporin induces the inhibition of ribosomes, prevents protein synthesis, and results in cell death. (**D**). Knockdown of CD320 using siRNA might help to inhibit the proliferation of CD320-overexpressing cells. Due to the decreased expression of CD320 and decreased uptake of VB12, cells would not be able to meet a high demand for DNA synthesis, which hopefully will result in the inhibition of cell proliferation. (**E**) It is possible to design a molecule directly targeting CD320 overexpressed in cells. The TCPP is a porphyrin used for diagnostic purposes that utilizes CD320 to enter cancer cells. Created in Biorender (https://app.biorender.com/illustrations/676e46b28e51ca549ec7af8e?slideId=05cebd01-cb70-40e6-9dbe-1fb1f585fc9f, accessed on 29 April 2025).

Despite the absence of existing treatments targeting CD320, different therapeutic and diagnostic methods involving CD320 are emerging. Tetra(4-carboxyphenyl)porphyrin, whose uptake is mediated via the CD320 receptor, has been used for the early classification of patients into lung cancer and high-risk groups via staining sputum samples with considerable accuracy [35]. The methodology was successfully tested at a clinical trial [36]. Furthermore, tumor imaging with a radiolabeled VB12 derivative (99m)Tc-PAMA-cobalamin in patients with metastatic cancer showed to be promising [37]. It highlights both the therapeutic and diagnostic potential of CD320 targeted cancer treatment. In this part of the review, several CD320 targeting drug delivery systems will be discussed. Described methods are summarized in Table 1 and Figure 4.

### 2.1. VB12 Conjugates-Based Drug Delivery

Water-solubility, absence of toxicity, and protection of protein–peptide drugs from degradation in acidic conditions by VB12 makes it a good carrier for cytotoxic agents (Figure 4) [6,31,52,53]. Even after conjugation with a drug, directly or via a spacer, VB12 is still recognized by internal transporters, including transcobalamin, allowing for the development of prodrugs and CD320-specific targeting [31].

#### 2.1.1. VB12–Platinum Conjugates

Several anticancer agents use metals as a base. For example, platinum is used widely in the development of anticancer agents, such as cisplatin, carboplatin, and oxaliplatin [54]. To resolve issues of poor bioavailability, non-specificity for tumors, and severe side effects of metal-based agents, their conjugates with carrier molecules were synthesized [31,54,55]. Due to the high demand for VB12 in cancer cells, it is possible that the conjugation of metal-based anticancer agents to VB12 can resolve the drawbacks of non-specific targeting and improve therapeutic outcomes.

In the study of Ruiz-Sánchez et al., the conjugation of cyanocobalamin, a derivative of VB12, to the cisplatin-like precursors via the cyano group resulted in the formation of the VB12–platinum complexes [6,56]. The formed conjugates were recognized by adenosyltransferase, resulting in the release of adenosylcobalamin and cytotoxic platinum–cyano complex inside the cell (Figure 4A) [6,56,57]. Unfortunately, in vitro studies showed that VB12–platinum complexes themselves are less potent compared to cisplatin [6].

Another attempt at the conjugation of drugs with VB12 via platinum bridge was conducted by Tran et al. [38]. The clinically approved drugs cytarabine, dacarbazine, and anastrozole were conjugated to VB12 via the {CN-*trans*-Pt(NH3)~2~} bridge [38]. The formed complex was shown to be a prodrug with a two-step activation mechanism. The interaction of VB12 with its transport proteins, including TC, was not altered upon attachment to the complex [38]. The release of the platinum complex and the active drug took place upon the reduction of central cobalt ion of VB12 inside the cell. However, similarly to the VB12–platinum complex described above, the prodrug was shown to be less potent compared to the used active drug.

Recently, the effect of fluorescent VB12–platinum conjugates was tested on prostate cancer (PC3), cervical carcinoma (HeLa), and breast cancer (MCF7) [39]. Unfortunately, obtained fluorescent conjugates showed little to no cytotoxicity on tested cells and did not affect the cell cycle of treated cells. However, as the authors claimed, the research represents the beginning of development of the VB12–platinum conjugates for cancer treatment.

In all cases, the expression of CD320 and the availability of apo-TC might serve as a limiting factor making VB12–platinum complexes less potent compared to corresponding unconjugated drugs. For example, the uptake of cisplatin-like derivatives was shown to be approximately three-folds lower compared to free cisplatin; this might also explain the lower cytotoxicity of the tested conjugates [31].

Some of the studies showed a higher accumulation of positively charged derivatives of VB12 inside the cell compared to neutral conjugates, which can be implemented for new VB12–metal derivative design [53,58].

The major concern in using these metal-based agents is the cytotoxicity to the liver and kidney. The liver and kidneys are used to store VB12 in normal conditions, and active drugs released into the intracellular space are cleared by these organs [11]. Haptocorrin-bound VB12 is recognized by asialoglycoprotein receptors of hepatocytes, while the renal absorption of VB12 is mediated via the megalin receptor recognizing TC-bound VB12 [22,59]. Both studies showed the ability of VB12–platinum conjugates to recognize and bind the transport proteins of VB12, raising concerns over liver and kidney toxicity. Thus, further studies on cancer specificity and liver, kidney, and other normal tissue cytotoxicity tests need to be conducted.

#### 2.1.2. VB12–Nitric Oxide Conjugates

Nitric oxide (NO) is a good anticancer agent, as intracellular NO inhibits cellular metabolism, induces DNA damage, and leads to apoptosis and necrosis [60,61,62,63,64]. The classical NO donors are non-selective to cancer cells, which results in damage to both normal and cancer cells [41]. Vitamin B12-based NO donors, such as nitrosylcobalamin, can potentially work as chemotherapeutic drugs (Figure 4A) [4,65].

The anti-tumor effect of nitrosylcobalamin was tested on four dogs with spontaneous cancer [40]. A long-term daily use of nitrosylcobalamin was shown to induce positive changes in tumor progression without causing systemic toxicity [40]. Bauer et al. claim that, in rodent and canine models, no bone marrow suppression, liver or kidney toxicity, and hypertension were observed upon nitrosylcobalamin administration, indicating an absence of systemic toxicity upon the delivery of nitrosylcobalamin [41]. NO release from nitrosylcobalamin was observed for acidic pH 5.0, while an insignificant release was shown at physiological conditions (pH 7.4), indicating that a major release takes place upon the internalization and formation of lysosome [4].

The high concentrations of NO (>1 μM) were shown to induce apoptosis via protein nitrosylation, including S-nitrosylation of a death receptor 4 (DR4) [66,67]. The overexpression of Apo2 ligands (tumor necrosis factor-related apoptosis-inducing ligands (Apo2L/TRAIL)), which is necessary for DR4-mediated cell death in tumor cells, but not in normal cells, further increases the tumor specificity of nitrosylcobalamin treatment [68].

Another possibility is to use nitrosylcobalamin in combination with other therapeutic agents. Most of the chemotherapeutic agents used to treat cancer cells activate NF-kB and Akt signaling, resulting in a decreased apoptotic potential of the drug, thus promoting cancer cells’ survival as well as the development of chemo-resistance [41,69,70,71,72]. In contrast, nitrosylcobalamin was shown to decrease the activation of both NF-kB and Akt signaling [73,74]. Thus, combining nitrosylcobalamin treatment with other chemotherapeutic drugs seems to be a promising strategy to prevent the development of drug resistance. Indeed, in 78% of the combinations of chemotherapeutic agents with nitrosylcobalamin, the synergistic anti-proliferative effect was observed in an in vitro study [41]. However, the extent of the inactivation of signaling cascades varied between different cells. As such, the combinations of treatment and cell type should be tested before application. In vivo studies showed that nitrosylcobalamin treatment results in tumor shrinkage and combination of nitrosylcobalamin and etoposide led to either tumor disappearance or tumor inhibition for 99% in tumor-bearing nude mice.

One concern regarding the usage of nitrosylcobalamin as a therapeutic agent is the identification of proper dosages, as at higher concentrations NO contributes to cancer progression [61,64]. Therefore, the administration of proper dosages per cell type, and the method of delivery, needs to be extensively studied.

#### 2.1.3. VB12–Colchicine Conjugate

Colchicine is a cytotoxic agent that prevents tubulin polymerization at low concentrations and microtubule depolymerization at higher concentrations [42,75]. Because of the overbearing systemic toxicity of colchicine, it was not used to treat cancer [42,76,77]. However, by conjugating it with cobalamin, it is possible to decrease untargeted cytotoxicity (Figure 4A). Colchicine conjugation to VB12 via acid-labile hydrazone stabilizes the complex at a neutral pH, which will prevent unwanted toxicity [42]. At physiological pH, it takes 9 days for the VB12–colchicine conjugate to undergo hydrolysis [42]. However, in a low pH environment of the lysosome, hydrazone is expected to undergo hydrolysis much faster, which would allow for the release of colchicine intracellularly [42]. Developed colchicine analogs had lower toxicity compared to pure colchicine when tested on neuroblastoma (SK-N-MC) and human breast adenocarcinoma (SK-BR-3) cells. However, the cytotoxicity of bioconjugates was approximately similar to chemotherapeutic drugs such as paclitaxel and docetaxel [42].

The targeted delivery of colchicine decreases its cytotoxicity and enables its usage for cancer treatment. However, the recognition of VB12–colchicine complexes via VB12 transport proteins and their effect on normal tissue, including liver and kidney tissues, need to be studied.

### 2.2. VB12 Conjugated Nanoparticles as a Drug Delivery System

Nanoparticles are widely used in biomedicine, as they are expected to improve the pharmacokinetics of the drugs [78]. They have a high drug-load capacity, can penetrate deep tissues, and have high retention and permeability, which makes them a desirable treatment delivery system [1,44,78]. Nanoparticles are used to deliver different types of therapeutics including mRNAs, small-interference RNAs, DNA, metals, and their complexes and chemotherapeutic agents [78]. Nanoparticle conjugation with VB12 would increase the specificity of targeting and enhance the chemotherapeutic agents’ delivery to cancer cells overexpressing the CD320 receptor (Figure 4B).

#### 2.2.1. VB12–Sericin Nanoparticle

Paclitaxel (PTX) is used as a front-line drug against gastric cancer. It inhibits the spindle function in tumor cells, which results in tumor cell death [44,79]. Despite the initial positive response, the development of drug resistance decreases PTX’s effectiveness [80,81]. Issues with tumor accessibility, and severe cytotoxicity, further limit its application as an anticancer treatment [80]. It was possible to improve the effect of PTX and decrease its cytotoxicity via conjugation with nanoparticles. Sericin is a widely used biocompatible and biodegradable nanoparticle, which is already employed for drug and gene delivery [43,82,83,84]. To selectively target cancer cells, sericin was conjugated with VB12 (Figure 4B). It resulted in the formation of synthetically obtained VB12-modified sericin-poly(gamma-benzyl-L-glutamate) (PBLG) micelles, which were further loaded with PTX [44]. Obtained micelles were confirmed to be biocompatible both in vitro and in vivo and they did not induce cell death in normal cells [44]. No inflammation and toxicity were observed when administered to Sprague-Dawley rats. VB12–sericin–PBLG–PTX treatment of BGC-823/PR gastric cancer cells induces early and total apoptosis three-fold higher compared to free PTX [44]. In vivo studies of VB12–sericin–PBLG–PTX treatment showed a 70% tumor growth reduction vs. 40% for a free PTX treatment in tumor-bearing nude mice [44]. It indicates improvement in the efficiency of drug delivery by VB12–sericin–PBLG nanoparticle. Histopathological and weight loss examination showed that VB12–sericin–PBLG–PTX is safe to administer [44]. Also, the CD320 receptor was confirmed to be responsible for the internalization of the micelle, indicating its potency as a cancer treatment target.

#### 2.2.2. PLGA-PEG-VB12 Nanoparticle

One of the possible targets for cancer treatment is the mitochondria of cancer cells. As highly proliferative cancer cells require a high energy input, they rely on the high metabolic function of mitochondria [85]. In addition, mitochondria are involved in apoptosis; therefore, by targeting the mitochondria of cancer cells, it is possible to inhibit cancer cell proliferation and induce apoptosis. One method of mitochondrial function regulation is microRNA (miRNA) [45]. miRNA is a class of small regulatory RNA, which binds to 3′-UTR (untranslated region) of mRNA and results in its degradation [86]. Researchers have specifically shown that miR-535-3p can increase the apoptosis rate in gastric cancer cells [87]. To stabilize and deliver miRNA to tumor sites, it is possible to use nanoparticles (Figure 4B). Polylactic-co-glycolic acid (PLGA) and polyethylene glycol (PEG) are biocompatible, biodegradable, attractive materials for the delivery of drugs and miRNAs [88,89,90].

PLGA-PEG nanoparticles conjugated with VB12 (PLGA-PEG-VB12) were loaded with miR-532-3p that targets apoptosis repressors with the caspase recruitment domain (ARC). PLGA-PEG-VB12 nanoparticle-embedded miRNA was shown to be stable, and the release of miRNA took place at pH 6.0. As the tumor microenvironment has a slightly acidic pH compared to a normal cell microenvironment, the release of miRNA at a slightly acidic pH can be considered an advantage of the developed system [45]. The targeting selectivity was confirmed, as the incubation of healthy gastric mucosa epithelial cells with PLGA-PEG-VB12 nanoparticles loaded with miR-532-3p did not show a significant induction of cell death [45]. In contrast, the same nanoparticles showed a higher inhibition of gastric cancer cell proliferation [45]. The uptake of miR-532-3p increased Bax expression in the mitochondria of BGC-823 cells (gastric cancer) and initiated apoptosis in these cells [45].

In vivo studies on BGC-823 cancer cells injected into tumor-bearing mice showed that, compared to the control group, the tumor growth was suppressed by almost 90% and 65% upon treatment with PLGA-PEG-VB12 and PLGA-PEG nanoparticles loaded with miR-532-3p, respectively, indicating advantages of VB12-based drug delivery systems [45]. Furthermore, as mice with PLGA-PEG-VB12 nanoparticles loaded with miR-532-3p experience only slight body weight changes, researchers came to a positive conclusion on the safety of the treatment. However, the effects of the developed nanoparticles were tested only on BGC-823 cancer cells that overexpress the CD320 receptor, and therefore it is not possible to conclude on the effectiveness of the treatment. Thus, further studies involving other cancers and normal cells need to be conducted.

#### 2.2.3. VB12 Conjugated with Stealth Liposome

Liposomes are one of the means of drug delivery [5,91]. Stealth liposomes and surface-modified liposomes are considered to be more potent drug carriers due to their ability to escape from the reticuloendothelial system, and due to their prolonged circulation time [5,92,93]. However, prolonged circulation results in cardiotoxicity, alopecia, and nausea, which limit their application [94]. Thus, the conjugation of stealth liposomes with ligands can improve their targeting ability and decrease their circulation time [5,93]. It is possible to conjugate a stealth liposome with VB12 (VB12-SL), to increase its selectivity for cancer cells overexpressing CD320.

The therapeutic potential of VB12-SL loaded with doxorubicin was tested on B16F10 cells (murine metastatic melanoma) [5]. Conjugation with VB12 did not affect the stability of the liposome. Cells incubated with sterically stabilized liposomes conjugated with VB12 showed 6.28-fold higher intracellular uptake of liposome content compared to normal sterically stabilized liposomes, illustrating that targeting is effective in vitro [5]. Similarly, the cytotoxicity was the strongest for VB12 conjugated stabilized liposomes compared to plain and unconjugated sterically stabilized liposomes.

C57BL/6 mice bearing B16F10 melanoma tumors were treated with doxorubicin containing VB12-SL. This treatment was able to effectively retard tumor growth compared to plain liposome, free doxorubicin, and saline controls [5]. The mean survival time of mice bearing primary tumors increased twice upon treatment with VB12-SL loaded with doxorubicin compared to the control treatment (free doxorubicin) [5]. The tissue distribution of doxorubicin after the intravenous injection of mice with doxorubicin loaded onto VB12-SL showed a lower distribution in the heart, liver, kidney, and spleen, compared to doxorubicin loaded in plain liposome and unconjugated stealth liposome [5]. However, the toxicity of doxorubicin in VB12-SL on these organs was not discussed. Thus, further tests need to be conducted.

### 2.3. CD320 Receptor Targeting Antibody and Nanobody Conjugates

Another method of targeting cells overexpressing CD320 is using monoclonal antibodies against CD320 conjugated with different agents (Figure 4C) [95,96]. It is possible to conjugate either primary or secondary antibodies to a drug or chemotherapeutic agent, which will selectively kill or induce senescence in cells. Furthermore, monoclonal antibodies against CD320 were already successfully implemented to induce VB12 deficiency in cancer cells [97,98]. As VB12 can be uptaken by asialoglycoprotein and megalin receptors in the liver and kidney, respectively, the conjugation of toxic agents to VB12 might induce undesired organ toxicity. In contrast, conjugating toxins to antibodies specific for CD320 is expected to not cause such side effects. However, as CD320 might be overexpressed in healthy, highly proliferative cells including stem cells, its targeting might lead to unwanted cytotoxicity.

#### 2.3.1. Saporin-Conjugated Antibodies Against CD320

One of the candidate drugs for antibody conjugation is saporin—a non-selective toxin that inactivates ribosomes and gains high specificity upon its conjugation with different ligands such as antibodies and peptides [99,100,101].

Three primary antibodies targeting the extracellular domain of CD320 were implemented (mAb 1-19, 1-23, 1-25) to test the effect of secondary antibodies conjugated with saporin on cancer cells [2,46]. The incubation of CD320 overexpressing HEK293 cells with these antibodies resulted in a more than 90% inhibition of cell growth in vitro [46]. The saporin-conjugated antibody treatment did not affect the growth of tested normal human fibroblasts (064, MCH065, RF). Bone marrow cells, however, experienced some proliferation inhibition, which serves as a point of concern [46]. Overall, the saporin conjugated to secondary antibodies increased the cell specificity of treatment.

To test the direct effect of saporin-conjugated monoclonal primary antibodies against CD320, three monoclonal antibodies (1-10, 1-19, 1-25) were constructed [47]. The average cell growth inhibition was similar for all three antibodies, despite targeting different CD320 domains [47]. In CD320-overexpressing HEK293 cells, a 2.5 nM concentration of monoclonal antibodies conjugated with saporin was enough to induce a 100% inhibition of proliferation, while in normal HEK293 cells, the same concentration of antibodies induced approximately 50% inhibition of cell proliferation, showing the selectivity of the antibodies [47]. Treatment with saporin-conjugated monoclonal antibodies against CD320 had minimal or no effect on the inhibition of normal cell proliferation (RFP3—skin fibroblast and ED—trophoblast cells) [47]. However, tested cancer cells showed different susceptibility to saporin-conjugated antibodies. Colorectal adenocarcinoma (PC-3, SW480), colon carcinoma and adenocarcinoma (RKO, Caco2), glioblastoma (U373), and epidermoid adenocarcinoma (KB) cells showed the highest cell proliferation inhibition, while breast carcinoma (MDA-MB-231) and colorectal adenocarcinoma (LoVo) were less susceptible to treatment. A possible explanation for the difference in the effect of antibody treatment might be the differences in the CD320 expression of tested cells.

Study outcomes suggest CD320 targeting via antibody-conjugated drugs as a potential cancer therapy, with an emphasis on discrimination between normal cells and highly proliferative cancer cells. However, follow-up studies involving normal cells and highly proliferative progenitors such as stem cells need to be conducted.

#### 2.3.2. Saporin-Conjugated Nanobodies Against TC

A nanobody is a heavy-chain fragment of an antibody, containing only one Ig domain [48,102]. The use of nanobodies is preferred over antibodies, due to their high stability, low immunogenicity, and ability to penetrate tissue (Figure 4C) [48,103]. Three saporin-conjugated nanobodies targeting the holo-TC-CD320 complex were shown to inhibit the growth of HEK293T cells, but to a different extent, e.g., TC-Nb4 nanobody conjugated to saporin had the strongest inhibitory effect on the growth of HEK293T cells. The difference in cytotoxicity might be due to the difference in affinity, as when compared to other nanobodies, TC-Nb4 had the highest affinity to both TC and CD320 [48]. Upon the treatment of highly proliferative HEK293T cells with 50 nM of TC-Nb4 nanobody conjugated with saporin, 100% cell death was observed. It is important to understand that scientists stimulated the expression of the *CD320* gene in HEK293T cells by seeding them at low density. Due to the high expression of CD320, nanobodies conjugated with saporin were able to inhibit cell growth. This means that highly proliferative normal cells might experience inhibition upon treatment with developed saporin-conjugated antibodies. As the effect of the nanobodies was tested on HEK293T cells only, further studies on cells with different CD320 expression need to be conducted.

### 2.4. Porphyrins as Potential Therapeutics Against Cancers with CD320 Overexpression

Another potential approach to target CD320-overexpressed cancer cells is to identify known and novel small-molecule anticancer agents and drugs, whose uptake depends on CD320 receptor (Figure 4E). Drugs from the porphyrin group could be potential candidates for this role. Porphyrins were identified to have an affinity to tissues with a high mitotic index. This phenomenon resulted in the development of cancer diagnostics (early lung and bladder cancer) and therapeutics for the treatment of lung cancer, localized prostate cancer, and bulk tumors, where porphyrins act as photosensitizers producing ROS in photodynamic therapy (PDT) and/or conjugates with cytotoxic anti-cancer drugs [35,104,105,106,107].

The mechanisms of porphyrins’ aggregation in tumor cells are not well elucidated. Still, it is believed that these mechanisms are based on porphyrins’ binding affinity to low-density lipoprotein receptors (LDLRs) overexpressed in many cancers, and entering tumor cells via passive diffusion with a high rate due to the low pH value of tumor tissues. Significantly, CD320 is a member of LDLR family, and meso-tetra (4-carboxyphenyl) porphyrin (TCPP), used for diagnostic purposes, was recently identified to bind, and internalize, via CD320 receptor-mediated endocytosis [49]. The knockdown of *CD320* in HCC15 (lung squamous cell carcinoma) and H157 (lung squamous cell carcinoma) cells resulted in 39% and 11% TCPP uptake inhibition, respectively. Similar results were obtained for prostate cancer cells (LNCaP—30% reduction, DU145—21% reduction) and breast cancer cells (MDA-MB-231—39% reduction). However, PC3 (prostatic adenocarcinoma), H358 (bronchoalveolar carcinoma), and MCF-7 (breast cancer) cells did not change their TCPP uptake upon the knockdown of *CD320* [49]. One possible explanation for the absence of a knockdown effect might be low CD320 expression in these cells. Another possible explanation is the presence of other receptors compensating for the knockdown of CD320.

Notably, porphyrins share a structural similarity with VB12. Overall, new porphyrin-based compounds with high specificity to CD320 could be potential candidates for new efficient and tumor-specific anti-cancer therapeutics against cancers with CD320 overexpression.

### 2.5. Knockdown of CD320 Using siRNA

Impaired VB12 metabolism, either by the depletion of VB12 or via the prevention of its internalization, was shown to reduce the proliferation of cancer cells [108]. Therefore, approaches to interrupt VB12 uptake via modification of CD320 expression might also be applied against cancer cell proliferation. Small-interference RNA (siRNA)-based knockdown seems a promising alternative method in the induction of VB12 deficiency (Figure 4D) [50,109].

siRNA knockdown of CD320 in HEK293 and SW48 (human colorectal adenocarcinoma) cells inhibited cell proliferation and increased the doubling time for both cell lines [50]. The high VB12 concentration in the culture medium did not restore the cells’ proliferation, pointing to the existence of only one mechanism of cellular VB12 uptake in tested cell lines [50].

Another study by Elzi et al. concluded that the simultaneous knockdown of two LDLR-family receptors, CD320 and LRP2, is necessary for the selective killing of cancer cells while discriminating against normal ones [51]. LRP2 is a receptor strongly expressed in the kidney which also internalizes the TC-VB12 complex [110]. In normal fibroblasts, simultaneous knockdown of these two genes did not affect the cells’ proliferation. However, in lung, breast, prostate, brain, and skin cancer cells, knockdown of both *CD320* and *LRP2* inhibited cell proliferation and induced the death of up to 80% of the cells [51]. In some cell lines, individual knockdown of either *CD320* or *LRP2* resulted in an increased expression of concurrent receptors, indicating the compensation effect of receptors [51]. Thus, the silencing of both receptors is essential for these cells.

CD320 knockdown seems to be a good therapeutic method that can be used upon the development of resistance to conventional therapies [50,109]. Due to the importance of VB12 metabolism, usage of siRNA against CD320 is less likely to induce resistance. However, due to the presence of different mechanisms for VB12 uptake for different cells, the strategies for the induction of VB12 deficiency via siRNA knockdown would be different.

## 3. Discussion

To selectively kill cancer cells without causing systemic toxicity and side effects, it is important to choose target proteins with high and low expression profiles in cancer and normal cells, respectively. This would allow for the development of new effective anticancer therapies and design of novel therapeutic agents acting as drug carriers and/or targeting upregulated cancer-specific proteins. One of the potential candidate proteins is CD320. CD320 involved in VB12 uptake is highly expressed in cancer cells while presenting a low expression profile in normal and differentiated cells (Figure 3) [1,2,4,5,6,7]. The overexpression of CD320 by cancer cells is likely caused by a high demand for VB12, which is necessary for DNA synthesis and more [10,11,12].

### 3.1. Biological Importance of CD320 and Future Directions

CD320 may serve as a good marker in the detection of different types of cancer and serve as a potential target of new anti-cancer treatments. Two studies involving osteosarcoma and hepatocellular carcinoma showed that CD320 plays an important role in cancer progression and proliferation. The study investigating the role of the tumor microenvironment in highly invasive osteosarcoma identified a high expression of *CD320* and *MAF* genes in tumor infiltrating regulatory T cells (Tregs) [32]. Based on these two genes, they were able to construct a prognostic risk model, which was shown to accurately predict osteosarcoma patients’ outcome [32]. Upregulated expression of CD320 was also observed for osteosarcoma tissue compared to adjacent normal tissues [32]. Another study identified CD320 as an early biomarker of hepatocellular carcinoma (HCC). CD320 was found to be positively associated with tumor microenvironment-forming cells (B cells, CD4^+^ T cells, macrophages, and dendritic cells) [7]. The survival and regression analysis showed that CD320 is a significant predictor and prognostic biomarker of HCC [7]. Both studies indicated that an increased expression of CD320, both in tumors and in tumor microenvironments, is essential for tumor progression. Thus, via targeting CD320, it would be possible to not only selectively kill cancer cells, but also target tumor microenvironments.

**Figure 5:**
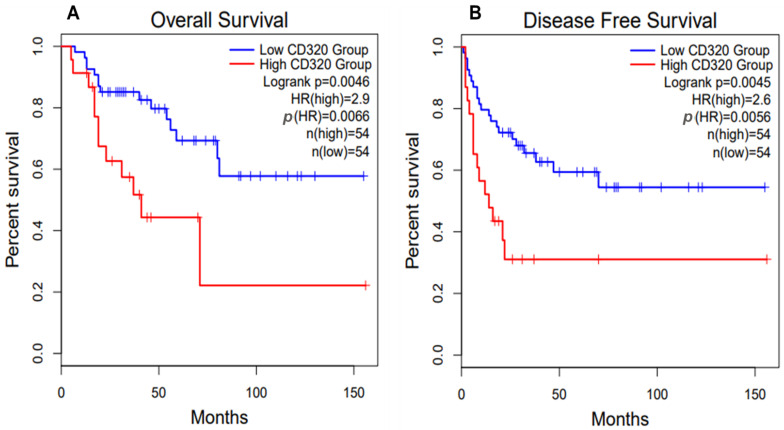
Survival plots for patients with adrenocortical carcinoma (ACC) with high and low CD320 expression. ACC patients with high expression of CD320 demonstrate poorer overall survival (**A**) and disease-free (**B**) survival. Data were retrieved from the Gene Expression Profiling Interactive Analysis 2 (GEPIA2) database [33,34]. The high and low cut-off values were set at 30% and 70%, respectively.

Based on data retrieved from Gene Expression Profiling Interactive Analysis 2 (GEPIA2) database, the high CD320 expression in patients with adrenocortical carcinoma is associated with poorer overall and disease-free survival (Figure 5) [33,34]. Therefore, similarly to HCC and osteosarcoma, CD320 might serve as a good prognostic marker and potential therapeutic target for adrenocortical carcinoma.

CD320 is expected to play an important role in the central nervous system (CNS). CD320 knockout mice experienced severe B12 depletion in the CNS, despite having normal levels of VB12 in the kidney and liver [111]. The knockout of CD320 in mice resulted in impaired peripheral sensation, demyelination, and other functional and structural changes of the central nervous system (anxiety, memory deficits) [112]. Moreover, analysis of serum and cerebrospinal fluid (CSF) of patients with tremor, ataxia, and scanning speech revealed the presence of patient-derived anti-CD320 antibodies in the patient samples, but not in the majority of healthy control samples [113]. The impairment of CD320 by anti-CD320 antibodies correlates with VB12 deficiency in the brain and the presence of neurologic deficits. This suggests that CD320 is the only receptor responsible for the VB12 uptake in the central nervous system [113]. However, further studies on the role of CD320 in the CNS and CD320 targeting of tumors of the CNS need to be conducted.

### 3.2. Limitations of Using CD320 as the Target for Cancer Treatment

For the application of CD320 targeting therapy, it is essential to first identify the responsiveness of different types of cancer and tissues to this treatment. Rhodamine-label lysine-modified hydroxypropyl methacrylic acid (HPMA) polymer conjugated with VB12 was shown to image different cancer cells to different extents in vitro [1]. For example, leukemic and ovarian mouse cells had low relative uptakes of HPMA-VB12 conjugate (L1210, Ov2008, ID8, Ovcar-3), while colon, lung, renal, and breast, cancer cells showed high uptake of the polymer (Colo-26, M109, RENCA, RD995, 4T1, JC, MMT060562) [1]. The difference in responses might be caused by the expression of CD320 receptors by specific cancer types. If true, then the cells would likely respond to the treatment to varying degrees depending on the level of CD320 expression (Figure 3).

An additional concern in targeting CD320 with VB12–cytotoxic drug conjugates is the existence of other pathways and receptors involved in the uptake of VB12 in cancer and normal cells. The simultaneous knockdown of *CD320* and *LRP2* is necessary to inhibit cancer cell growth, indicating the presence of other pathways involved in VB12 uptake [51]. Furthermore, due to the high demand for VB12, aggressive cancers might develop different methods of VB12 uptake [52]. For example, increased haptocorrin expression was observed for cancers such as breast, kidney, colon, lung, and pancreatic [18,52,114]. These cancer cells might have developed new pathways of VB12 uptake via haptocorrin rather than transcobalamin and CD320 receptor uptake. Thus, before the treatment administration, the effect on different cancers needs to be tested first.

Another issue of using the VB12 conjugates to target CD320 overexpressing cancer cells is that of the accumulation of drugs and chemotherapeutic agents in the kidneys and liver due to VB12 uptake by these organs [1]. Therefore, CD320-specific antibodies or porphyrins conjugated with cytotoxic drugs could be a safer choice of anti-cancer therapies. Then, most of the studies involving CD320 targeting tested the effect of developed complexes on cells only. Further in vivo studies testing the safety and efficiency of CD320 targeted therapy need to be performed.

Despite promising outcomes presented by potential anti-cancer treatment strategies based on CD320 targeting, they need to be tested on different types of cancer first. The different factors such as TC and CD320 expression levels and the presence of any alternative pathways of VB12 uptake are expected to affect the treatment outcome.

## 4. Conclusions

To conclude, CD320 targeting seems to be a promising strategy in cancer treatment, which prevents the death of healthy cells. It is possible to target CD320 overexpressing cells using VB12-conjugated agents and nanoparticles, antibodies, and nanobodies conjugated with drugs specific for CD320. However, as most of the studies of derivatives targeting CD320 were performed only on cell cultures, it is not possible to conclude on their effective discrimination between healthy and cancerous cells. Further in vivo studies testing the effects of treatment on the liver, kidney, highly proliferative stem cells, and other healthy cells need to be performed. Moreover, depending on the expression of CD320, different cancer cells show an inhibition of proliferation to different extents. Thus, it is essential to test the effect of CD320-targeted therapy on different cancers first.

## Figures and Tables

**Table 1:** Summary of described approaches of targeting CD320-overexpressing cancer cells.

CD320 Targeting Methods	Models	Outcomes	References
VB12–Platinum conjugates	In vitro—MCF and A2780 cells	Release of platinum from VB12–platinum conjugate took place inside the cell; VB12–platinum derivatives are less potent compared to cisplatin; Availability of VB12 receptor—CD320 might serve as a limiting factor.	[6]
	In vitro—K562 cells	Release of the cytotoxic drug from VB12–platinum–drug complexes took place inside the cell; VB12–platinum–cytarabine derivatives are less potent than cytarabine.	[38]
	In vitro—PC3, HeLa, MCF7 cells	Fluorescent VB12–platinum derivatives showed no/little cytotoxicity in vitro.	[39]
VB12-nitric oxide conjugates	In vivo—canine models	Long-term, daily use of the nitrosylcobalamin conjugates resulted in tumor volume reduction and tumor regression in dogs; No bone marrow suppression, liver, and kidney cytotoxicity were observed in treated animals.	[40]
	In vitro—13 malignant cancer cells In vivo—nude mice	Nitrosylcobalamin prevents drug resistance by inhibiting NF-kB and Akt signaling; 78% of nitrosylcobalamin and other drug combinations result in inhibition of cancer cell proliferation; Nitrosylcobalamin treatment led to tumor shrinkage in vivo; Nitrosylcobalamin and etoposide combination treatment resulted in either tumor disappearance or tumor inhibition for 99% in vivo.	[41]
VB12–colchicine conjugate	In vitro—SK-N-MC, SK-BR-3 cells	Release of colchicine takes place in the lysosome, due to low pH; VB12–colchicine conjugates show cytotoxicity lower than free colchicine, but similar to paclitaxel and docetaxel.	[42]
VB12–sericin–PBLG nanoparticle loaded with paclitaxel	In vitro—red blood cells, MCF10A, LO2), RAW264.7, MCF7, HepG2 cells In vivo—Sprague-Dawley rats, nude mice	VB12–sericin–PBLG nanoparticles show no cytotoxicity or inflammation when administered to rats; Doxorubicin loaded to VB12–sericin–PBLG nanoparticle induces apoptosis more compared to free paclitaxel; Treatment of tumor-bearing mice with nanoparticles loaded with paclitaxel resulted in tumor growth suppression; No weight loss, histological abnormalities of heart, liver, spleen, lung, kidneys, brain tissues were observed for treated rats; VB12–sericin–PBLG nanoparticle-based delivery of paclitaxel allowed for gastric cancer targeting and more efficient induction of apoptosis.	[43,44]
PLGA-PEG-VB12 nanoparticles	In vitro—BGC-823, GES-1 cells In vivo—nude mice	PLGA-PEG-VB12 nanoparticles are shown to be biocompatible and cancer-specific; PLGA-PEG-VB12 nanoparticles loaded with miR-532-3p induced apoptosis in gastric cancer cells via activation of ARC/Bax/mitochondria-mediated apoptosis cell signaling; VB12-conjugated nanoparticles loaded with miRNA showed tumor growth suppression in vivo.	[45]
VB12–stealth liposome loaded with doxorubicin	In vitro—B16F10 In vivo—C57BL/6 mice	Loading of doxorubicin onto VB12-stealth liposome increased drug’s half-life; VB12–stealth liposomes accumulate in tumor tissues two-fold higher compared to control stealth liposomes; Treatment with VB12–stealth liposomes loaded with doxorubicin prolongs the survival time of mice compared to free doxorubicin administration.	[5]
Saporin-conjugated antibodies against CD320	In vitro—K562, U266, SW48, KB and HEK293 cells, normal human fibroblasts, bone marrow cells	The effect of saporin-conjugated secondary antibody treatment depended on CD320 expression of tested cells; The inhibitor effect of saporin-conjugated secondary antibodies is tumor-specific; Low cell proliferation inhibition is observed for bone marrow cells.	[46]
	In vitro—K562, SW48, HL60, U266, RKO, HeLa, LoVo, HepG2, Hep3B, MDA-MB-231, PaCa-2U373, PC3, MCH064, MCH065, RF, bone marrow mononuclear cells, human chorionic trophoblasts, ED, HEK293 cells	Saporin-conjugated primary antibodies did not interfere with CD320-TC-VB12 complex internalization; The effect of saporin-conjugated primary antibody treatment depends on CD320 expression of tested cells; Saporin-conjugated primary antibody treatment inhibited the cell proliferation of cancer cells to approximately 50%; No/minimal inhibition was observed for normal cells.	[47]
Saporin-conjugates nanobodies against TC	In vitro—HEK293T cells	The affinity of saporin-conjugated nanobodies to TC-VB12 complex correlated with their ability to induce cell death in vitro; Saporin-conjugated nanobodies induced 100% cell death of CD320 expressing HEK293T cells.	[48]
meso-tetra (4-carboxyphenyl) porphyrin (TCPP)	In vitro—HCC15, H157, LNCaP, DU145, MDA-MB-231, PC3, H358, MCF7 cells	TCPP was identified to enter the cell via CD320 receptor binding; Knockdown of CD320 in different cancer cells affects the TCPP uptake differently.	[49]
siRNA knockdown of *CD320*	In vitro—HEK293, SW48 cells	Knockdown of *CD320* in HEK239 and SW48 inhibited cell proliferation and increased the doubling time; The effect of knockdown seems to depend on the expression of CD320.	[50]
	In vitro—normal fibroblasts, lung, brain, breast, prostate, skin cancer cells	*CD320* and *LRP2* knockdown is essential for selective cancer cytotoxicity; Knockdown of both genes did not affect normal fibroblasts and induced up to 80% cell death in cancer cells.	[51]

## Abbreviations

The following abbreviations are used in this manuscript:

VB12	vitamin B12
Cbl	cobalamin
MS	methionine synthase
MCM	methylmalonylCoA mutase
TCA cycle	tricarboxylic acid
ATP	adenosine triphosphate
MRP1	multidrug resistance-associated protein 1
TCblR	transcobalamin II receptor
LDLR	low-density lipoprotein receptor
TC-VB12	transcobalamin–vitamin B12 complex
TC	transcobalamin
MZF1	Myeloid Zinc Finger 1
RREB1	Ras-responsive element-binding protein 1
C/EBP	CCAAT-enhancer-binding proteins
HNF-3beta	hepatocyte nuclear factor 3-beta
AP-1	activating protein-1
GFP	green fluorescent protein
dsRed	red fluorescent protein
NO	nitric oxide
DR4	death receptor
PTX	paclitaxel
SL	stealth liposome
TCPP	4-carboxyphenyl porphyrin
PLGA	polylactic-co-glycolic acid
PEG	polyethylene glycol
HPMA	hydroxypropyl methacrylic acid
ACC	adrenocortical carcinoma

## References

[1] Russell-Jones G., McTavish K., McEwan J., Rice J., Nowotnik D. (2004). Vitamin-mediated targeting as a potential mechanism to increase drug uptake by tumours. J. Inorg. Biochem..

[2] Quadros E.V., Nakayama Y., Sequeira J.M. (2009). The protein and the gene encoding the receptor for the cellular uptake of transcobalamin-bound cobalamin. Blood.

[3] Alam A., Woo J.S., Schmitz J., Prinz B., Root K., Chen F., Bloch J.S., Zenobi R., Locher K. (2016). Structural basis of transcobalamin recognition by human CD320 receptor. Nat. Commun..

[4] Bauer J.A. (1998). Synthesis, characterization and nitric oxide release profile of nitrosylcobalamin. Anti-Cancer Drugs.

[5] Gupta Y., Ganesh N., Kohli D.V., Jain S.K. (2011). Development and characterization of doxorubicin bearing vitamin B12 coupled sterically stabilized liposomes for tumor targeting. Curr. Nanosci..

[6] Ruiz-Sánchez P., König C., Ferrari S., Alberto R. (2011). Vitamin B12 as a carrier for targeted platinum delivery: In vitro cytotoxicity and mechanistic studies. J. Biol. Inorg. Chem..

[7] Lin X.H., Li D.P., Liu Z.Y., Zhang S., Tang W.Q., Chen R.X., Weng S.Q., Tseng Y.J., Xue R.Y., Dong L. (2023). Six immune-related promising biomarkers may promote hepatocellular carcinoma prognosis: A bioinformatics analysis and experimental validation. Cancer Cell Int..

[8] Zhang S., Jiang Z., Wang P., Jiang W., Ding W., Zhong L. (2024). TCBIR/CD320: A potential therapeutic target upregulated in endothelial cells and associated with immune cell infiltration in liver hepatocellular carcinoma. Discov. Oncol..

[9] Quadros E.V. (2010). Advances in the understanding of cobalamin assimilation and metabolism. Br. J. Haematol..

[10] Smith A.D., Warren M.J., Refsum H. (2018). Vitamin B12. Adv. Food Nutr. Res..

[11] Nielsen M.J., Rasmussen M.R., Andersen C.B.F., Nexø E., Moestrup S.K. (2012). Vitamin B12 transport from food to the body’s cells—A sophisticated, multistep pathway. Nat. Rev. Gastroenterol. Hepatol..

[12] Chen Y., Gu X., Zhang Y., Zhang X., Zhang C., Liu M., Sun S., Dong N. (2022). CD320 expression and apical membrane targeting in renal and intestinal epithelial cells. Int. J. Biol. Macromol..

[13] Quadros E.V., Sequeira J.M. (2013). Cellular uptake of cobalamin: Transcobalamin and the TCblR/CD320 receptor. Biochimie.

[14] Stabler S.P., Allen R.H. (2004). Vitamin B12 deficiency as a worldwide problem. Annu. Rev. Nutr..

[15] Haghighat G., Khajeh-Mehrizi A., Ranjbar H. (2023). Evaluation of serum vitamin B12 levels in patients with colon and breast cancer: A case-control study. Int. J. Hematol. Oncol. Stem Cell Res..

[16] Sottotetti F., Malovini A., Maccarone S., Riva G., Tibollo V., Palumbo R., Tagliaferri B., Bellazzi R., Cena H., Di Sabatino A. (2024). Vitamin B12 status in hospitalised cancer patients: Prevalence and clinical implications of depletion and hypervitaminosis. Clin. Nutr. ESPEN.

[17] Hall C.A. (1975). Transcobalamins I and II as natural transport proteins of vitamin B12. J. Clin. Investig..

[18] Burger R.L., Schneider R.J., Mehlman C.S., Allen R.H. (1975). Human plasma R type vitamin B12 binding proteins. II. The role of transcobalamin I, transcobalamin III, and the normal granulocyte vitamin B12 binding protein in the plasma transport of vitamin B12. J. Biol. Chem..

[19] Rothenberg S.P., Weiss J.P., Cotter R. (1978). Formation of transcobalamin II-vitamin B12 complex by guinea-pig ileal mucosa in organ culture after in vivo incubation with intrinsic factor- vitamin B12. Br. J. Haematol..

[20] Jiang W., Nakayama Y., Sequeira J.M., Quadros E.V. (2013). Mapping the functional domains of TCblR/CD320, the receptor for cellular uptake of transcobalamin-bound cobalamin. FASEB J..

[21] Quadros E.V., Regec A.L., Khan K.M.F., Quadros E., Rothenberg S.P. (1999). Transcobalamin II synthesized in the intestinal villi facilitates transfer of cobalamin to the portal blood. Am. J. Physiol. Gastrointest. Liver Physiol..

[22] Moestrup S.K., Birn H., Fischer P.B., Petersen C.M., Verroust P.J., Sim R.B., Christensen E.I., Nexø E. (1996). Megalin-mediated endocytosis of transcobalamin-vitamin-B12 complexes suggests a role of the receptor in vitamin-B12 homeostasis. Proc. Natl. Acad. Sci. USA.

[23] Quadros E.V., Nakayama Y., Sequeira J.M. (2005). The binding properties of the human receptor for the cellular uptake of vitamin B12. Biochem. Biophys. Res. Commun..

[24] Gick G.G., Arora K., Sequeira J.M., Nakayama Y., Lai S.C., Quadros E.V. (2020). Cellular uptake of vitamin B12: Role and fate of TCblR/CD320, the transcobalamin receptor. Exp. Cell Res..

[25] Hall C.A. (1984). The uptake of vitamin B12 by human lymphocytes and the relationships to the cell cycle. J. Lab. Clin. Med..

[26] Hall C.A., Colligan P.D., Begley J.A. (1987). Cyclic activity of the receptors of cobalamin bound to transcobalamin II. J. Cell. Physiol..

[27] Lindemans J., Kroes A.C.M., Geel J.V., Van Kapel J., Schoester M., Abels J. (1989). Uptake of transcobalamin II-bound cobalamin by HL-60 cells: Effects of differentiation induction. Exp. Cell Res..

[28] Amagasaki T., Green R., Jacobsen D.W. (1990). Expression of transcobalamin II receptors by human leukemia K562 and HL-60 cells. Blood.

[29] Jiang W., Sequeira J.M., Nakayama Y., Lai S.C., Quadros E.V. (2010). Characterization of the promoter region of TCblR/CD320 gene, the receptor for cellular uptake of transcobalamin-bound cobalamin. Gene.

[30] Takahashi K., Tavassoli M., Jacobsen D.W. (1980). Receptor binding and internalization of immobilized transcobalamin II by mouse leukaemia cells. Nature.

[31] Pettenuzzo A., Pigot R., Ronconi L. (2016). Vitamin B12–metal conjugates for targeted chemotherapy and diagnosis: Current status and future prospects. Eur. J. Inorg. Chem..

[32] Cheng D., Zhang Z., Mi Z., Tao W., Liu D., Fu J., Fan H. (2023). Deciphering the heterogeneity and immunosuppressive function of regulatory T cells in osteosarcoma using single-cell RNA transcriptome. Comput. Biol. Med..

[33] Tang Z., Li C., Kang B., Gao G., Li C., Zhang Z. (2017). GEPIA: A web server for cancer and normal gene expression profiling and interactive analyses. Nucleic Acids Res..

[34] Tang Z., Kang B., Li C., Chen T., Zhang Z. (2019). GEPIA2: An enhanced web server for large-scale expression profiling and interactive analysis. Nucleic Acids Res..

[35] Patriquin L., Merrick D.T., Hill D., Holcomb R.G., Lemieux M.E., Bennett G., Karia B., Rebel V.I., Bauer T. (2015). Early detection of lung cancer with meso tetra (4-carboxyphenyl) porphyrin-labeled sputum. J. Thorac. Oncol..

[36] Lemieux M.E., Reveles X.T., Rebeles J., Bederka L.H., Araujo P.R., Sanchez J.R., Grayson M., Lai S.C., DePalo L.R., Habib S.A. (2023). Detection of early-stage lung cancer in sputum using automated flow cytometry and machine learning. Respir. Res..

[37] Sah B.R., Schibli R., Waibel R., von Boehmer L., Bläuenstein P., Nexo E., Johayem A., Fischer E., Müller E., Soyka J.D. (2014). Tumor imaging in patients with advanced tumors using a new (99m) Tc-radiolabeled vitamin B12 derivative. J. Nucl. Med. Off. Publ..

[38] Tran M.T.Q., Furger E., Alberto R. (2013). Two-step activation prodrugs: Transplatin mediated binding of chemotherapeutic agents to vitamin B12. Org. Biomol. Chem..

[39] Mehder R., de la Torre-Rubio E., de la Cueva-Alique I., O’Malley C., Pérez-Redondo A., Gude L., Royo E., Ronconi L. (2024). Fluorescent Vitamin B_12_–Platinum(II) Derivatives as Potential Metallotheranostic Agents for the Treatment and Imaging of Tumors. Inorganics.

[40] Bauer J.A., Frye G., Bahr A., Gieg J., Brofman P. (2010). Anti-tumor effects of nitrosylcobalamin against spontaneous tumors in dogs. Investig. New Drugs.

[41] Bauer J.A., Lupica J.A., Schmidt H., Morrison B.H., Haney R.M., Masci R.K., Lee R.M., DiDonato J.A., Lindner D.J. (2007). Nitrosylcobalamin potentiates the anti-neoplastic effects of chemotherapeutic agents via suppression of survival signaling. PLoS ONE.

[42] Bagnato J.D., Eilers A.L., Horton R.A., Grissom C.B. (2004). Synthesis and characterization of a cobalamin-colchicine conjugate as a novel tumor-targeted cytotoxin. J. Org. Chem..

[43] Guo W., Deng L., Yu J., Chen Z., Woo Y., Liu H., Li T., Lin T., Chen H., Zhao M. (2018). Sericin nanomicelles with enhanced cellular uptake and pH-triggered release of doxorubicin reverse cancer drug resistance. Drug Deliv..

[44] Guo W., Deng L., Chen Z., Chen Z., Yu J., Liu H., Li T., Lin T., Chen H., Zhao M. (2019). Vitamin B12-conjugated sericin micelles for targeting CD320-overexpressed gastric cancer and reversing drug resistance. Nanomedicine.

[45] Chen Z., Liang Y., Feng X., Liang Y., Shen G., Huang H., Chen Z., Yu J., Liu H., Lin T. (2021). Vitamin-B12-conjugated PLGA-PEG nanoparticles incorporating miR-532-3p induce mitochondrial damage by targeting apoptosis repressor with caspase recruitment domain (ARC) on CD320-overexpressed gastric cancer. Mater. Sci. Eng. C.

[46] Quadros E.V., Nakayama Y., Sequeira J.M. (2010). Targeted delivery of saporin toxin by monoclonal antibody to the transcobalamin receptor, TCblR/CD320. Mol. Cancer Ther..

[47] Quadros E.V., Nakayama Y., Sequeira J.M. (2013). Saporin conjugated monoclonal antibody to the transcobalamin receptor TCblR/CD320 is effective in targeting and destroying cancer cells. J. Cancer Ther..

[48] Bloch J.S., Sequeira J.M., Ramírez A.S., Quadros E.V., Locher K.P. (2022). Generation of nanobodies targeting the human, transcobalamin-mediated vitamin B12 uptake route. FASEB J..

[49] Elzi D.J., Bauta W.E., Sanchez J.R., Das T., Mogare S., Zannes Fatland P., Iza M., Pertsemlidis A., Rebel V.I. (2021). Identification of a novel mechanism for meso-tetra (4-carboxyphenyl) porphyrin (TCPP) uptake in cancer cells. FASEB J..

[50] Lai S.C., Nakayama Y., Sequeira J.M., Quadros E.V. (2011). Down-regulation of transcobalamin receptor TCblR/CD320 by siRNA inhibits cobalamin uptake and proliferation of cells in culture. Exp. Cell Res..

[51] Elzi D.J., Bauta W., Lai S., Das T., Mogare S., Rebel V.I. Simultaneous knockdown of CD320 and LRP2 receptors is selectively toxic to cancer cells but not normal cells [Poster abstract]. Proceedings of the American Association of Cancer Research (AACR) Annual Meeting 2021.

[52] Waibel R., Treichler H., Schaefer N.G., Van Staveren D.R., Mundwiler S., Kunze S., Küenzi M., Alberto R., Nüesch J., Knuth A. (2008). New derivatives of vitamin B12 show preferential targeting of tumors. Cancer Res..

[53] Tran M.T., Stürup S., Lambert I.H., Gammelgaard B., Furger E., Alberto R. (2016). Cellular uptake of metallated cobalamins. Met. Integr. Biometal Sci..

[54] Kelland L. (2007). The resurgence of platinum-based cancer chemotherapy. Nat. Rev. Cancer.

[55] McWhinney S.R., Goldberg R.M., McLeod H.L. (2009). Platinum neurotoxicity pharmacogenetics. Mol. Cancer Ther..

[56] Mundwiler S., Spingler B., Kurz P., Kunze S., Alberto R. (2005). Cyanide-bridged vitamin B12-cisplatin conjugates. Chemistry.

[57] Ruiz-Sánchez P., Mundwiler S., Spingler B., Buan N.R., Escalante-Semerena J.C., Alberto R. (2008). Syntheses and characterization of vitamin B12-Pt(II) conjugates and their adenosylation in an enzymatic assay. J. Biol. Inorg. Chem..

[58] Mellman I., Willard H.F., Youngdahl-Turner P., Rosenberg L.E. (1979). Cobalamin coenzyme synthesis in normal and mutant human fibroblasts. Evidence for a processing enzyme activity deficient in cblC cells. J. Biol. Chem..

[59] Alpers D., Russell-Jones G., Banerjee R. (1999). Intrinsic factor, haptocorrin and their receptors. Chemistry and Biochemistry of B12.

[60] Burney S., Caulfield J.L., Niles J.C., Wishnok J.S., Tannenbaum S.R. (1999). The chemistry of DNA damage from nitric oxide and peroxynitrite. Mutat. Res..

[61] Ramírez-Patiño R., Avalos-Navarro G., Figuera L.E., Varela-Hernández J.J., Bautista-Herrera L.A., Muñoz-Valle J.F., Gallegos-Arreola M.P. (2022). Influence of nitric oxide signaling mechanisms in cancer. Int. J. Immunopathol. Pharmacol..

[62] Änggård E. (1994). Nitric oxide: Mediator, murderer, and medicine. Lancet.

[63] Gross S.S., Wolin M.S. (1995). Nitric oxide: Pathophysiological mechanisms. Annu. Rev. Physiol..

[64] Alimoradi H., Greish K., Gamble A.B., Giles G.I. (2019). Controlled delivery of nitric oxide for cancer therapy. Pharm. Nanotechnol..

[65] Dunphy M.J., Sysel A.M., Lupica J.A., Griffith K., Sherrod T., Bauer J.A. (2014). A stability-indicating HPLC method for the determination of nitrosylcobalamin (NO-Cbl), A novel vitamin B12 analog. Chromatographia.

[66] Tang Z., Bauer J.A., Morrison B., Lindner D.J. (2006). Nitrosylcobalamin promotes cell death via S nitrosylation of Apo2L/TRAIL receptor DR4. Mol. Cell. Biol..

[67] Mannick J.B., Schonhoff C.M. (2004). NO means no and yes: Regulation of cell signaling by protein nitrosylation. Free Radic. Res..

[68] Ashkenazi A., Pai R.C., Fong S., Leung S., Lawrence D.A., Marsters S.A., Blackie C., Chang L., McMurtrey A.E., Hebert A. (1999). Safety and antitumor activity of recombinant soluble Apo2 ligand. J. Clin. Investig..

[69] Baldwin A.S. (2001). Control of oncogenesis and cancer therapy resistance by the transcription factor NF-κB. J. Clin. Investig..

[70] Kim D., Chung J. (2002). Akt: Versatile mediator of cell survival and beyond. J. Biochem. Mol. Biol..

[71] Wang C.Y., Cusack J.C., Liu R., Baldwin A.S. (1999). Control of inducible chemoresistance: Enhanced anti-tumor therapy through increased apoptosis by inhibition of NF-κB. Nat. Med..

[72] Khwaja A. (1999). Apoptosis: Akt is more than just a bad kinase. Nature.

[73] Matthews J.R., Botting C.H., Panico M., Morris H.R., Hay R.T. (1996). Inhibition of NF-κB DNA binding by nitric oxide. Nucleic Acids Res..

[74] Størling J., Binzer J., Andersson A.K., Züllig R.A., Tonnesen M., Lehmann R., Spinas G.A., Sandler S., Billestrup N., Mandrup-Poulsen T. (2005). Nitric oxide contributes to cytokine-induced apoptosis in pancreatic beta cells via potentiation of JNK activity and inhibition of Akt. Diabetologia.

[75] Leung Y.Y., Yao Hui L.L., Kraus V.B. (2015). Colchicine—Update on mechanisms of action and therapeutic uses. Semin. Arthritis Rheum..

[76] Bhattacharyya B., Panda D., Gupta S., Banerjee M. (2008). Anti-mitotic activity of colchicine and the structural basis for its interaction with tubulin. Med. Res. Rev..

[77] Iacobuzio-Donahue C.A., Lee E.L., Abraham S.C., Yardley J.H., Wu T.T. (2001). Colchicine toxicity: Distinct morphologic findings in gastrointestinal biopsies. Am. J. Surg. Pathol..

[78] Yusuf A., Almotairy A.R.Z., Henidi H., Alshehri O.Y., Aldughaim M.S. (2023). Nanoparticles as drug delivery systems: A review of the implication of nanoparticles’ physicochemical properties on responses in biological systems. Polymers.

[79] Zhu L., Chen L. (2019). Progress in research on paclitaxel and tumor immunotherapy. Cell. Mol. Biol. Lett..

[80] Dan V.M., Raveendran R.S., Baby S. (2020). Resistance to intervention: Paclitaxel in breast cancer. Mini-Rev. Med. Chem..

[81] Smith E.R., Wang J.Q., Yang D.H., Xu X.X. (2022). Paclitaxel resistance related to nuclear envelope structural sturdiness. Drug Resist. Updates.

[82] Huang L., Tao K., Liu J., Qi C., Xu L., Chang P., Gao J., Shuai X., Wang G., Wang Z. (2016). Design and fabrication of multifunctional sericin nanoparticles for tumor targeting and pH-responsive subcellular delivery of cancer chemotherapy drugs. ACS Appl. Mater. Interfaces.

[83] Mandal B.B., Kundu S.C. (2009). Self-assembled silk sericin/poloxamer nanoparticles as nanocarriers of hydrophobic and hydrophilic drugs for targeted delivery. Nanotechnology.

[84] Crivelli B., Perteghella S., Bari E., Sorrenti M., Tripodo G., Chlapanidas T., Torre M.L. (2018). Silk nanoparticles: From inert supports to bioactive natural carriers for drug delivery. Soft Matter.

[85] Zhu Y., Dean A.E., Horikoshi N., Heer C., Spitz D.R., Gius D. (2018). Emerging evidence for targeting mitochondrial metabolic dysfunction in cancer therapy. J. Clin. Investig..

[86] Hayes J., Peruzzi P.P., Lawler S. (2014). MicroRNAs in cancer: Biomarkers, functions and therapy. Trends Mol. Med..

[87] Guo W., Chen Z., Chen Z., Yu J., Liu H., Li T., Lin T., Chen H., Zhao M., Li G. (2018). Promotion of Cell Proliferation through Inhibition of Cell Autophagy Signalling Pathway by Rab3IP is Restrained by MicroRNA-532-3p in Gastric Cancer. J. Cancer.

[88] Danhier F., Ansorena E., Silva J.M., Coco R., Le Breton A., Préat V. (2012). PLGA-based nanoparticles: An overview of biomedical applications. J. Control. Release.

[89] Chen X., Chen J., Li B., Yang X., Zeng R., Liu Y., Li T., Ho R.J.Y., Shao J. (2017). PLGA-PEG-PLGA triblock copolymeric micelles as oral drug delivery system: In vitro drug release and in vivo pharmacokinetics assessment. J. Colloid Interface Sci..

[90] Veronese F.M., Pasut G. (2005). PEGylation, successful approach to drug delivery. Drug Discov. Today.

[91] Samad A., Sultana Y., Aqil M. (2007). Liposomal drug delivery systems: An update review. Curr. Drug Deliv..

[92] Čeh B., Winterhalter M., Frederik P.M., Vallner J.J., Lasic D.D. (1997). Stealth^®^ liposomes: From theory to product. Adv. Drug Deliv. Rev..

[93] Immordino M.L., Dosio F., Cattel L. (2006). Stealth liposomes: Review of the basic science, rationale, and clinical applications, existing and potential. Int. J. Nanomed..

[94] English J.C., Toney R., Patterson J.W. (2003). Intertriginous epidermal dysmaturation from pegylated liposomal doxorubicin. J. Cutan. Pathol..

[95] Polito L., Bolognesi A., Tazzari P.L., Farini V., Lubelli C., Zinzani P.L., Ricci F., Stirpe F. (2004). The conjugate rituximab/saporin-S6 completely inhibits clonogenic growth of CD20-expressing cells and produces a synergistic toxic effect with fludarabine. Leukemia.

[96] Baah S., Laws M., Rahman K.M. (2021). Antibody–drug conjugates—A tutorial review. Molecules.

[97] Quadros E.V., McLoughlin P., Rothenberg S.P. (1996). Characterization of monoclonal antibodies to epitopes of human transcobalamin II. Biochem. Biophys. Res. Commun..

[98] McLean G.R., Quadros E.V., Rothenberg S.P., Morgan A.C., Schrader J.W., Ziltener H.J. (1997). Antibodies to transcobalamin II block in vitro proliferation of leukemic cells. Blood.

[99] Bolshakov A.P., Stepanichev M.Y., Dobryakova Y.V., Spivak Y.S., Markevich V.A. (2020). Saporin from Saponaria officinalis as a tool for experimental research, modeling, and therapy in neuroscience. Toxins.

[100] Polito L., Bortolotti M., Mercatelli D., Battelli M.G., Bolognesi A. (2013). Saporin-S6: A useful tool in cancer therapy. Toxins.

[101] Yadav S., Batra J. (2015). Mechanism of anti-HIV activity of ribosome inactivating protein, saporin. Protein Pept. Lett..

[102] Muyldermans S. (2021). Applications of nanobodies. Annu. Rev. Anim. Biosci..

[103] De Meyer T., Muyldermans S., Depicker A. (2014). Nanobody-based products as research and diagnostic tools. Trends Biotechnol..

[104] Lange N., Jichlinski P., Zellweger M., Forrer M., Marti A., Guillou L., Kucera P., Wagnières G., Van Den Bergh H. (1999). Photodetection of early human bladder cancer based on the fluorescence of 5-aminolaevulinic acid hexylester-induced protoporphyrin IX: A pilot study. Br. J. Cancer.

[105] Anand S., Ortel B.J., Pereira S.P., Hasan T., Maytin E.V. (2012). Biomodulatory approaches to photodynamic therapy for solid tumors. Cancer Lett..

[106] Zhang Q., He J., Yu W., Li Y., Liu Z., Zhou B., Liu Y. (2020). A promising anticancer drug: A photosensitizer based on the porphyrin skeleton. RSC Med. Chem..

[107] Xue X., Lindstrom A., Li Y. (2019). Porphyrin-Based Nanomedicines for Cancer Treatment. Bioconjugate Chem..

[108] Battaglia-Hsu S.-F., Akchiche N., Noel N., Alberto J.-M., Jeannesson E., Orozco-Barrios C.E., Martinez-Fong D., Daval J.-L., Guéant J.-L. (2009). Vitamin B12 deficiency reduces proliferation and promotes differentiation of neuroblastoma cells and up-regulates PP2A, proNGF, and TACE. Proc. Natl. Acad. Sci. USA.

[109] Ashihara E., Kawata E., Maekawa T. (2010). Future prospect of RNA interference for cancer therapies. Curr. Drug Targets.

[110] Renard E., Chéry C., Oussalah A., Josse T., Perrin P., Tramoy D., Voirin J., Klein O., Leheup B., Feillet F. (2019). Exome sequencing of cases with neural tube defects identifies candidate genes involved in one-carbon/vitamin B12 metabolisms and sonic hedgehog pathway. Hum. Genet..

[111] Lai S.C., Nakayama Y., Sequeira J.M., Wlodarczyk B.J., Cabrera R.M., Finnell R.H., Bottiglieri T., Quadros E.V. (2013). The transcobalamin receptor knockout mouse: A model for vitamin B12 deficiency in the central nervous system. FASEB J..

[112] Arora K., Sequeira J.M., Hernández A.I., Alarcon J.M., Quadros E.V. (2017). Behavioral alterations are associated with vitamin B12 deficiency in the transcobalamin receptor/CD320 KO mouse. PLoS ONE.

[113] Pluvinage J.V., Ngo T., Fouassier C., McDonagh M., Holmes B.B., Bartley C.M., Kondapavulur S., Hurabielle C., Bodansky A., Pai V. (2024). Transcobalamin receptor antibodies in autoimmune vitamin B12 central deficiency. Sci. Transl. Med..

[114] Gimsing P., Hippe E. (1978). Increased concentration of transcobalamin I in a patient with metastatic carcinoma of the breast. Scand. J. Haematol..

